# Supplementary material for: Determinants of skilled birth attendant delivery among reproductive age women in Lesotho: A multilevel analysis of demographic and health survey data
Source: PLoS One. 2026 Jan 6;21(1):e0340011. doi: 10.1371/journal.pone.0340011 (PMC12774358; doi:10.1371/journal.pone.0340011)
Supplement: Attachment — Submitted filename: Response to Reviewers.docx [file pone.0340011.s002.docx]

Date: October 07, 2025

PLOS ONE

Manuscript tittle: Determinants of skilled birth attendant delivery among reproductive age women in Lesotho: a multilevel analysis.

Submission ID: PONE-D-25-34001

**Response to Journal Requirements:**

1. Please ensure that your manuscript meets PLOS ONE's style requirements, including those for file naming.

**Authors’ response:** We have ensured that our manuscript aligns with PLOS ONE's style requirements, following the templates provided. The revised version adheres to the required formatting and file-naming conventions.

2. We note that your Data Availability Statement is currently as follows: [All relevant data are within the manuscript and its Supporting Information files]. Please confirm at this time whether or not your submission contains all raw data required to replicate the results of your study.

**Authors’ response:** We confirm that all relevant data required to replicate the study findings are included in the manuscript and supporting information files. We will provide them or deposit the dataset in a public repository as recommended.

3. Please include captions for your Supporting Information files at the end of your manuscript, and update any in-text citations to match accordingly.

**Authors’ response:** Thank you for this suggestion. We have added caption for the Supporting Information file at the end of the manuscript.

4. We are unable to open your Supporting Information file [SBA.DTA]. Please kindly revise as necessary and re-upload.

**Authors’ response:** Thank you for bringing this to our attention. The Supporting Information file [SBA.DTA] is a Stata dataset file, which requires Stata software to open. We have re-checked the file and confirmed that it opens correctly in Stata. The file has been re-uploaded and renamed as **Lesotho 2023-24 DHS data.DTA** to ensure it can be accessed without any issues.

5. If the reviewer comments include a recommendation to cite specific previously published works, please review and evaluate these publications to determine whether they are relevant and should be cited. There is no requirement to cite these works unless the editor has indicated otherwise.

**Authors’ response:** Thank you for bringing this to our attention. We note that no specific publications were suggested for citation, and therefore no additional references were added.

**Point by point response to Reviewers’ comments**

**Reviewer #1:**

**Comment 1:** The title seems like for the case-control, I recommend a little bit to modify

**Authors’ response:** Thank you for your valuable comment. We have revised the title to accurately reflect the study design: as “Determinants of skilled birth attendant delivery among reproductive age women in Lesotho: a multilevel analysis of demographic and health survey data”

**Comment 2:** List the abbreviation of SBA when you use in the first line of the sentences (Line 38 & 233) and similar comment throughout the paper. In this section mention the AOR for the factors positively associated with SBA. In the recommendation what are the targeted polices (it is general, but the other recommended ideas are good).

**Authors’ response:** Thank you for your insightful comment. We have defined “SBA” at first use and throughout the manuscript. AORs for statistically significant predictors have been added in the abstract. The recommendations were revised to include specific actions.

**Comment 3:** Although the authors highlighted several important points in the introduction, they failed to adequately address the issue of maternal death and the SBA, particularly by omitting recent evidence and trends from global to regional (Africa) levels. Also cite the reference for Line 63-64 (for the maternal death in Lesotho).

**Authors’ response:** Thank you for your comment. We expanded the Introduction to include updated statistics on maternal mortality and SBA trends globally, in sub-Saharan Africa, and in Lesotho. We also cite the references for line 63–64.

**Comment 4:** Change “methods and Materials” to Method; In the operational definition I recommend changing “Community Poverty Level” to Economic status or other word; the media exposure is also not clear for readers. Change “Data management and analysis” to Data analysis. Change “Ethics approval and consent to participants” to Ethical consideration

**Authors’ response:** Thank you very much for your constructive comments. We tried to incorporate the comment in the manuscript.

**Comment 5:**  I recommend categorizing into background and reproductive health related characteristics separately with subhead instead of “descriptive characteristics”.

**Authors’ response:** Thank you for your comment. We tried to incorporate the comment in the manuscript.

**Comment 6:** In the discussion of the magnitude of skilled birth attendant (SBA) utilization, comparisons with both higher and lower prevalence rates reported in other studies should be presented and briefly discussed. These comparisons should include logical justifications for the observed differences relative to the current findings. For instance, in Line 175, only a lower prevalence was mentioned, and the cited reference lacks clarity, making it difficult for readers to interpret the comparison effectively. The discussion in the factors section is well-articulated; however, there are a few minor points that require attention. Specifically, I recommend the authors include the AOR for each factor discussed to provide clear information to readers. Additionally, the study location should be mentioned for each referenced study. For example, in Line 180, references 28 and 29, the geographical area where each study was conducted should be specified. I recommend Similar revisions for Line 210, Line 212, and other sections where contributing factors are discussed

**Authors’ response:** Thank you for this valuable comment. We have revised the discussion to compare our findings with available published studies and included possible explanations for the observed differences. Since there were no studies reporting higher SBA prevalence in comparable contexts, we compared our findings with those reporting similar or lower prevalence rates. Adjusted odds ratios (AORs) for each discussed factor were also incorporated to enhance clarity. Each cited reference now clearly specifies the country or region of origin to improve clarity and context.

**Comment 7:** “Addressing these challenges through targeted policies and programs is essential. Strategies aimed at improving healthcare accessibility, availability, and financial support should be prioritized to enhance SBA coverage and ultimately improve maternal health outcomes.”
The statement is general; it would be more effective to recommend specific actions based on the findings.

**Authors’ response:** Thank you for your valuable comment. We revised the Conclusion to provide actionable recommendations based on our findings.

**Comment 8:** Table 1: The number of participants is 1407 but for the variables Age, marital status, Media exposure, health insurance coverage, SBA, community level media exposure is 1406. Distance to health facility: what do you mean ‘Big problem or No big problem”
What is the difference between media exposure and community level media exposure in this paper?

**Authors’ response:** Thank you for your comment. The minor inconsistency in sample size occurred because we used weighted frequencies, which produced decimals; these have now been rounded to the nearest whole number. The variable Distance to health facility has been clarified to reflect the respondent’s self-reported perception of whether distance to a health facility posed a major problem in accessing maternal healthcare services. Additionally, the operational definitions of media exposure (individual-level exposure to radio, television, or newspapers) and community-level media exposure (the aggregated proportion of women in the community reporting media exposure) have been clearly specified in the Methods section.

**Reviewer #2**:

**Reviewers’ comment:** Thank you for this excellent paper. It has analysed recent data resulting in succinct and readable paper with appropriate conclusions. You have speculated on some of the possible reasons behind the findings. This is important because it is only the answers to those questions of "why did a certain group act in a certain way" that can provide real understanding and lead to necessary interventions. I would have suggested that in the conclusion you might have alluded to a need for more detailed qualitative research to be conducted to further elucidate this point, but this is not a criticism. I rarely find that I can make a recommendation for a paper to be accepted without alteration but in this case I feel this recommendation to accept is well justified.

**Authors’ response:** We sincerely thank the reviewer for the thoughtful and positive feedback. We greatly appreciate the recognition of the clarity, readability, and relevance of our analysis. We also value your suggestion regarding the potential for further qualitative research to explore the underlying reasons behind our findings. To address this, we have included a sentence in the Conclusion highlighting the need for more detailed qualitative studies to better understand the factors influencing skilled birth attendant utilization. We are grateful for your recommendation to accept our manuscript.
