# Supplementary material for: Determinants of skilled birth attendant delivery among reproductive age women in Lesotho: A multilevel analysis of demographic and health survey data
Source: PLoS One. 2026 Jan 6;21(1):e0340011. doi: 10.1371/journal.pone.0340011 (PMC12774358; doi:10.1371/journal.pone.0340011)
Supplement: Attachment — Submitted filename: Response_to_Reviewers_auresp_2.docx [file pone.0340011.s003.docx]

Date: December 10, 2025

PLOS ONE

Manuscript tittle: Determinants of skilled birth attendant delivery among reproductive age women in Lesotho: a multilevel analysis of demographic and health survey data.

Submission ID: PONE-D-25-34001

**Response to Journal Requirements:**

1. If the reviewer comments include a recommendation to cite specific previously published works, please review and evaluate these publications to determine whether they are relevant and should be cited. There is no requirement to cite these works unless the editor has indicated otherwise.

**Authors’ response:** Thank you for bringing this to our attention. We note that no specific publications were suggested for citation, and therefore no additional references were added.

2. Please review your reference list to ensure that it is complete and correct. If you have cited papers that have been retracted, please include the rationale for doing so in the manuscript text, or remove these references and replace them with relevant current references. Any changes to the reference list should be mentioned in the rebuttal letter that accompanies your revised manuscript. If you need to cite a retracted article, indicate the article’s retracted status in the References list and also include a citation and full reference for the retraction notice.

**Authors’ response:** Thank you for this important comment. We have thoroughly reviewed the entire reference list to ensure accuracy, completeness, and compliance with the journal’s guidelines. During this review, we confirmed that none of the cited references have been retracted. Therefore, no retracted articles were included, and no changes to the reference list were required. Accordingly, no additional citations were added or removed.

**Point by point response to Reviewers’ comments**

**Reviewer #3:**

**Comment 1:** The study does not sufficiently highlight what is new compared to previous evidence. Authors should clearly identify how this analysis adds to the existing body of knowledge on SBA utilization in Lesotho.

**Authors’ response:** Thank you for this valuable comment. We have now strengthened the Introduction sections to more clearly articulate the novelty of our study.

**Comment 2:** The global and country-level maternal mortality figures are inconsistent and somewhat confusing (e.g., 566.2 vs. 529 per 100,000). Clarify whether these are WHO estimates and ensure consistency between the abstract and introduction. Define “healthcare professionals” and ensure comparability with the operational definition of SBA. The rationale should be reframed to justify the study in light of persistently high MMR despite high SBA coverage.

**Authors’ response:** Thank you for this comment. We have corrected and aligned the maternal mortality figures in both the abstract and introduction using the latest WHO estimates. We also clarified the definition of “healthcare professionals” to ensure consistency with the WHO-defined operational definition of skilled birth attendants. Additionally, we revised the rationale to emphasize the persistent gap between high SBA coverage and the still elevated maternal mortality ratio in Lesotho, highlighting the need for updated evidence from the 2023–2024 LSDHS.

**Comment 3:** Clearly describe why random effect analysis was used and mention it explicitly in the methodology. Explain the rationale for including specific variables (e.g., community-level indicators). Ensure consistency in terminology (e.g., “Economic status” vs. “Community poverty level”).

**Authors’ response:** Thank you for these helpful suggestions. We have now explicitly stated in the methodology that a random-effects (multilevel) analysis was used to account for the hierarchical structure of the DHS data, where individuals are nested within clusters, and to capture unobserved community-level variation in SBA utilization. We also clarified the rationale for including specific community-level variables, noting that they provide important contextual information that may influence access, behaviours, and health service utilization beyond individual characteristics. In addition, we reviewed the manuscript to ensure consistent use of terminology, particularly standardizing the term “economic status.”

**Comment 4:** Present rural–urban differences, as this was not visible in the results despite being an important determinant. Ensure figures and tables have consistent sample sizes (1407 vs. 1406). Clarify variable meanings (e.g., “distance to health facility: big problem/no big problem”).

**Authors’ response:** Thank you for these comments. Upon review, we found that rural–urban residence did not show a meaningful difference in SBA utilization and was not significantly associated with the outcome. We also rechecked the tables and confirmed that there are no discrepancies in the reported sample size. Additionally, the meaning of variables such as “distance to health facility” is now clearly defined in the operational definitions section.

**Comment 5:**  Focus on key findings with policy or programmatic relevance to Lesotho rather than reiterating general determinants. Some comparative interpretations are contradictory (e.g., Nepal results cited with conflicting conclusions). The discussion overemphasizes descriptive comparison and lacks deeper explanation of why these associations persist.

**Authors’ response:** Thank you for these constructive comments. We have focused our discussion on key findings with policy and programmatic relevance to Lesotho and have provided interpretations based on the available data, while acknowledging the limitations inherent in secondary data analysis

**Comment 6:** Recommendations are too general. Since SBA coverage is already high, emphasize addressing equity and quality gaps. Consider aligning conclusions with findings, avoiding overly broad or repetitive policy statements. Inclusion of a recommendation for future qualitative research is appropriate and should be expanded slightly.

**Authors’ response:** Thank you for this insightful comment. We have revised the recommendations to focus on addressing equity and quality gaps in SBA utilization, given that coverage is already high in Lesotho. Conclusions have been aligned more closely with the study findings, avoiding overly broad or repetitive policy statements. Additionally, we have expanded the recommendation for future qualitative research to better understand the underlying barriers and facilitators of SBA utilization, including sociocultural, household, and health system factors.

**Comment 7:** Ensure consistent abbreviation use. Improve wording clarity (e.g., “area,” “domain,” “healthcare professionals”). Check grammar, punctuation, and spacing (comma vs. hyphen use for example in CI in abstract). Ensure data access and ethics sections meet PLOS ONE requirements precisely.

**Authors’ response:** Thank you for this comment. We have carefully reviewed the manuscript to ensure consistent use of abbreviations throughout. Wording has been clarified for terms such as “area,” “domain,” and “healthcare professionals,” and grammar, punctuation, and spacing including the presentation of confidence intervals in the abstract have been corrected. We also ensured that the data access and ethics statements fully comply with PLOS ONE requirements.

**Reviewer #4**:

**Reviewers’ comment**: The authors have adequately addressed the comments provided by the previous reviewers. However, the use of abbreviations can be improved, starting from page 1.

**Authors’ response:** We sincerely thank the reviewer for the thoughtful and positive feedback. We have carefully reviewed the manuscript and improved the use of abbreviations throughout.
